# The E3 Ubiquitin Ligase NEDD4L Targets OGG1 for Ubiquitylation and Modulates the Cellular DNA Damage Response

**DOI:** 10.3389/fcell.2020.607060

**Published:** 2020-11-12

**Authors:** Jonathan R. Hughes, Jason L. Parsons

**Affiliations:** ^1^Cancer Research Centre, Department of Molecular and Clinical Cancer Medicine, University of Liverpool, Liverpool, United Kingdom; ^2^Clatterbridge Cancer Centre NHS Foundation Trust, Bebington, United Kingdom

**Keywords:** DNA damage, DNA repair, OGG1, NEDD4L, ubiquitin

## Abstract

8-Oxoguanine DNA glycosylase (OGG1) is the major cellular enzyme required for the excision of 8-oxoguanine DNA base lesions in DNA through the base excision repair (BER) pathway, and therefore plays a major role in suppressing mutagenesis and in controlling genome stability. However, the mechanism of regulation of cellular OGG1 protein, particularly in response to oxidative stress, is unclear. We have purified the major E3 ubiquitin ligase responsible for OGG1 ubiquitylation from human cell extracts, and identify this as E3 ubiquitin-protein ligase NEDD4-like (NEDD4L). We demonstrate that recombinant NEDD4L stimulates ubiquitylation of OGG1 *in vitro*, particularly on lysine 341, and that NEDD4L and OGG1 interact in U2OS cells. Depletion of NEDD4L in U2OS cells has no impact on the stability and steady-state protein levels of OGG1, however, OGG1 stability is enhanced in response to oxidative stress induced by ionizing radiation. Furthermore, ubiquitylation of OGG1 by NEDD4L *in vitro* inhibits its DNA glycosylase/lyase activity. As a consequence of prolonged OGG1 stability and increased excision activity in the absence of NEDD4L, cells display increased DNA repair capacity but conversely that this decreases cell survival post-irradiation. This effect can be reproduced following OGG1 overexpression, suggesting that dysregulation of OGG1 increases the formation of lethal intermediate DNA lesions. Our study therefore highlights the importance of balancing OGG1 protein levels and BER capacity in maintaining genome stability.

## Introduction

Highly reactive oxygen species (ROS) are produced in cells as a consequence of oxidative metabolism, and in addition to exogenous sources including ionizing radiation (IR), these are able to attack DNA and disrupt genome integrity. Therefore, the formation of oxidative DNA base damage, base loss (apurinic/apyrimidinic or AP sites) and DNA strand breaks is a common occurrence (∼10,000 DNA lesions per cell per day; Lindahl, 1993). If the DNA damage is not resolved by the cellular DNA repair machinery, this can promote mutagenesis and lead to development of several human diseases, including premature aging, neurodegenerative diseases, and cancer. Fortunately, cells are equipped with damage-specific DNA glycosylases that excise DNA base modifications (Jacobs and Schar, 2012;Wallace, 2013), as well as other protein members of the base excision repair (BER) pathway that restore the DNA to its undamaged form. These proteins include AP endonuclease 1 (APE1) that incises the AP site formed following DNA glycosylase incision, DNA polymerase β (Pol β) that inserts the correct nucleotide and removes the 5′-deoxyribosephosphate moiety, and DNA ligase IIIα-X-ray repair cross-complementing protein 1 (Lig IIIα-XRCC1) complex that seals the nick in the DNA backbone (Parsons and Dianov, 2013).

Proteins within the BER pathway have been shown to be regulated by a number of post-translational modifications, including acetylation, phosphorylation and ubiquitylation, that control the efficiency of the pathway and therefore of the cellular DNA damage response (Carter and Parsons, 2016). In particular, ubiquitylation on site-specific lysine residues within BER proteins catalyzed by E3 ubiquitin ligases have been demonstrated to play a key role in controlling cellular steady state levels of proteins, as well as those in response to DNA damage stress (Edmonds and Parsons, 2014). For example, we have previously identified that Mcl-1 ubiquitin ligase E3 (Mule) and tripartite motif 26 (TRIM26) target the DNA glycosylase endonuclease VIII-like protein 1 (NEIL1) for ubiquitylation-dependent degradation which is important for controlling cell survival in response to IR (Edmonds et al., 2017). More recently, we have also shown that TRIM26 controls the cellular protein levels of another DNA glycosylase, endonuclease III homologue (NTH1), in response to oxidative stress induced by hydrogen peroxide (Williams and Parsons, 2018). Therefore, it is clearly evident that BER proteins, and the pathway itself, is tightly regulated by ubiquitylation in response to the endogenous levels of DNA damage.

8-Oxoguanine DNA glycosylase (OGG1) is the major DNA glycosylase that excises 8-oxoguanine lesions from DNA (Boiteux and Radicella, 2000), which is a well-known premutagenic lesion contributing to GC to TA transversions. OGG1 knockout mice have been shown to exhibit accumulation of 8-oxoguanine but only display moderate, but significant, spontaneous mutation rates (Klungland et al., 1999; Minowa et al., 2000; Sakumi et al., 2003), likely due to redundancy with other DNA glycosylases (e.g., NEIL1). However, reduced OGG1 protein, and thus activity, has been observed in prostate cancer cells (Trzeciak et al., 2004), and reduced OGG1 has also been associated with increased risk of head and neck squamous cell carcinoma (Paz-Elizur et al., 2006; Kumar et al., 2012) and with an aggressive form of breast cancer (Karihtala et al., 2012) observed utilizing patient tumor samples. Conversely, increased OGG1 protein expression and activity has been observed in colorectal cancer tissues (Kondo et al., 2000). Interestingly in TK6 lymphoblast cells, depletion of OGG1 caused reduced sensitivity to IR, whereas OGG1 overexpression enhanced cell death (Yang et al., 2004; Yang et al., 2006), suggesting that the balance of the protein is critical for controlling cell survival post-irradiation. Additionally, a well-characterized single nucleotide polymorphism of OGG1 (serine 326 to cysteine) is associated with a higher risk of developing a number of different cancers (Karahalil et al., 2012), due to deficiencies in the repair of 8-oxoguanine (Bravard et al., 2009; Kershaw and Hodges, 2012). More recently, it has been demonstrated that OGG1 plays an important role in preventing accumulation of telomeric 8-oxoguanine, and therefore telomere loss, vital for promoting cell growth (Fouquerel et al., 2019). Furthermore, OGG1 has been shown to have a role in controlling gene expression (Wang et al., 2018), specifically by excising 8-oxoguanine lesions from guanine-rich promotor sequences leading to promotion of G-quadruplex structures and subsequent transcriptional activation (Fleming et al., 2017). Collectively, it is clear that OGG1 is vital in the suppression of the levels of 8-oxoguanine in genomic DNA, and that the protein should be tightly regulated to prevent genome instability and mutagenesis that promote disease development. To this effect, it has been previously demonstrated that OGG1 is subject to ubiquitylation-dependent degradation by the E3 ubiquitin ligase carboxy terminus of Hsc70 interacting protein (CHIP), but only under conditions of mild hyperthermia (Fantini et al., 2013). Therefore, the specific E3 ubiquitin ligase enzymes and mechanisms that control the cellular protein levels of OGG1 particularly in response to oxidative DNA damage, are currently unknown.

Here, we have purified and identified E3 ubiquitin-protein ligase NEDD4-like (NEDD4L) as the major cellular E3 ubiquitin ligase that catalyzes ubiquitylation of OGG1 *in vitro*, and that accurate control of cellular OGG1 protein levels and activity are required for modulating overall BER capacity but also promoting cell survival in response to IR.

## Materials and Methods

### Reagents

OGG1 antibodies (ab124741) were from Abcam (Cambridge, United Kingdom), actin antibodies were from Sigma-Aldrich (Gillingham, United Kingdom), NEDD4L and TRIM21 antibodies were from Bethyl Laboratories (Montgomery, United States). HeLa cell pellets for protein fractionation by column chromatography were from Cilbiotech (Mons, Belgium). Ubiquitin was purchased from Boston Biochemicals (Cambridge, United States). Bacterial expression plasmids for E1 (UBE1) and 9 × E2 enzymes (UBCH2, UBCH3, UBCH5A, UBCH5B, UBCH5C, UBCH6, UBCH7, UBCH8, and UBCH10), as well as the mammalian expression plasmid for HA-tagged NEDD4L were acquired from Addgene (Cambridge, United States). Full length *ogg1* cDNA was re-cloned using ligation independent cloning (Aslanidis and De Jong, 1990) from a bacterial expression plasmid (pET28a) for OGG1 and into pCMV-Tag3a vector for mammalian expression. Conversely *nedd4l* cDNA was re-cloned into pET28a vector for bacterial expression. The *trim21* cDNA was also recloned into the same vector, using the mammalian expression plasmid kindly provided by Prof A. Garcia-Sastre. Site-directed PCR mutagenesis was used to generate site-specific mutants within OGG1 and catalytically inactive (C942A) NEDD4L. His-tagged NEDD4L, OGG1, E1, and E2 enzymes were overexpressed in Rosetta2(DE3)pLysS bacterial cells (Merck-Millipore, Watford, United Kingdom) and purified using HisTrap column chromatography (GE Healthcare, Little Chalfont, United Kingdom).

### Cell Culture, siRNA Knockdowns and Clonogenic Assays

U2OS cells (kindly provided by Prof G. Dianov) were cultured at 37°C in 5% CO_2_ in Dulbecco’s Modified Eagle Medium (DMEM) containing 10% fetal bovine serum, 2 mM L-glutamine, 1× penicillin–streptomycin and 1× non-essential amino acids. Cells were routinely tested to ensure absence of mycoplasma infection. For siRNA knockdowns, cells were cultured in 35 mm dishes for 24 h to 30–50% confluence and then treated with 2 μl Lipofectamine RNAiMAX transfection reagent (Life Technologies, Paisley, United Kingdom) in the presence of 40 nM Qiagen AllStars Negative Control siRNA (Qiagen, Southampton, United Kingdom), NEDD4L siRNA#1 (5′-GGAGACAGCAUUCUAUUUA-3′) or NEDD4L siRNA#2 (5′-GAAUAUCGCUGGAGACUCU-3′) for a further 72 h. For clonogenic assays, cells were irradiated in 35 mm dishes with the CellRad x-ray irradiator (Faxitron Bioptics, Tucson, United States), trypsinized, counted and a defined number seeded in triplicate into six-well plates and incubated at 37°C in 5% CO_2_. Note that double the numbers of cells were seeded following NEDD4L siRNA, and increasing cell numbers were used for increasing doses of x-ray irradiation to account for cellular plating efficiencies. Colonies were allowed to grow for 7–10 days, prior to fixing and staining with 6% glutaraldehyde, 0.5% crystal violet for 30 min. Plates were washed, left to air dry overnight and colonies counted using the GelCount colony analyzer (Oxford Optronics, Oxford, United Kingdom). Relative colony formation (surviving fraction) was expressed as colonies per treatment level versus colonies that appeared in the untreated control. Statistical analysis was performed using the CFAssay for R package (Braselmann et al., 2015).

### Whole Cell Extract Preparation, Cell Fractionation and Immunoprecipitations

Cells were harvested and whole cell extracts prepared as previously described (Edmonds et al., 2017; Williams and Parsons, 2018). Cell fractionation generating soluble and chromatin bound protein fractions was also performed as previously described (Edmonds et al., 2017). For immunoprecipitations, Protein A magnetic beads (New England Biolabs, Hitchin, United Kingdom) were washed three times with Buffer A [50 mM Tris–HCl (pH 8.0), 1 mM EDTA, 5% glycerol] using a magnetic separation rack and beads (10 μl) subsequently incubated with 0.5 μg OGG1 antibodies for 3 h at 4°C with shaking. OGG1 antibody-beads, along with a beads only control (mock immunoprecipitation), were washed three times with Buffer A and then incubated with 100 μg U2OS whole cell extracts for 2 h at 4°C with shaking. The immunodepleted extracts were removed from the beads, and the beads washed three times with Buffer A containing 150 mM KCl. SDS-PAGE sample buffer was added to the washed beads and heated for 5 min at 95°C prior to SDS-PAGE and immunoblotting. Proteins were separated by 10 % Tris-glycine SDS-PAGE, transferred onto an Immobilon FL PVDF membrane (Millipore, Watford, United Kingdom), blocked using Odyssey blocking buffer (Li-cor Biosciences, Cambridge, United Kingdom) and incubated with the primary antibody diluted in Odyssey blocking buffer with 0.1% Tween 20 overnight at 4°C. Membranes were washed three times with PBS containing 0.1% Tween 20 (5 min washes), incubated with either Alexa Fluor 680 or IR Dye 800-conjugated secondary antibodies for 1 h at room temperature and further washed three times with PBS containing 0.1% Tween 20. After a final wash with PBS, proteins were visualized and quantified using the Odyssey image analysis system (Li-cor Biosciences, Cambridge, United Kingdom).

### Purification of the E3 Ubiquitin Ligase From HeLa Whole Cell Extracts

HeLa whole cell extracts were prepared from 20 g HeLa cell pellets, as described above, and were dialyzed against Buffer A (50 mM Tris-HCl (pH 8.0), 5% glycerol, 1 mM EDTA, 1 mM DTT and 100 μM PMSF) containing 150 mM KCl. The cell extract was clarified by centrifugation (25,000 rpm for 20 min), filtered through 0.45 μm syringe filters, added to a 250 ml P-11 Phosphocellulose column and the flow-through collected (designated PC150). The PC150 fraction was diluted two-fold with Buffer A (achieving a final concentration of 75 mM KCl) and then added to a 20 ml HiLoad Mono Q Sepharose column (GE Healthcare, Little Chalfont, United Kingdom). The column was washed with Buffer A containing 50 mM KCl, proteins were eluted into 4 ml fractions using a 400 ml linear gradient from 50 to 1000 mM KCl and active fractions were then pooled and concentrated using Amicon Ultra-15 centrifugal filter units (Millipore, Watford, United Kingdom). Proteins were loaded onto a Superdex 200 HR 10/30 column (GE Healthcare, Little Chalfont, United Kingdom) in Buffer A containing 150 mM KCl and 0.5 ml fractions collected. Active fractions were pooled, concentrated and buffer exchanged using Amicon Ultra-15 centrifugal filter units into Buffer B [5 mM KPO_4_ (pH 7.0), 5 % glycerol, 1 mM DTT and 100 μM PMSF]. Proteins were applied to a 1 ml CHT ceramic hydroxyapatite column (Bio-Rad, Hemel Hempstead, United Kingdom) in Buffer B, and eluted into 0.5 ml fractions using a linear gradient of 5–500 mM KPO_4_. Active fractions were pooled, diluted 10-fold in Buffer A and then loaded onto a Mono Q 5/50 GL column (GE Healthcare, Little Chalfont, United Kingdom) in buffer A containing 50 mM KCl and proteins eluted into 0.5 ml fractions using a linear gradient of 50–1000 mM KCl. After each chromatography stage, protein fractions were examined for *in vitro* OGG1 ubiquitylation activity and those displaying significant activity were pooled for the next chromatography step. Proteins present in active fractions from the final Mono Q chromatography were identified by tandem mass spectrometry using the Q Exactive instrument operated in data dependent positive (ESI+) mode, as recently described (Edmonds et al., 2017).

### *In vitro* Ubiquitylation Assay and Immunoblotting

Ubiquitylation reactions (typically 15 μl) containing 6 pmol His-OGG1, 0.7 pmol GST-E1 activating enzyme, 2.5 pmol E2 conjugating enzyme (combination of nine different E2s, unless otherwise stated) and 0.6 nmol ubiquitin in buffer containing 25 mM Tris–HCl (pH 8.0), 4 mM ATP, 5 mM MgCl_2_, 200 μM CaCl_2_, and 1 mM DTT were incubated in LoBind protein tubes (Eppendorf, Stevenage, United Kingdom) for 1 h at 30°C with agitation. Reactions were halted by the addition of SDS-PAGE sample buffer [25 mM Tris–HCl (pH 6.8), 2.5% β-mercaptoethanol, 1% SDS, 10% glycerol, 1 mM EDTA, 0.05 mg/ml bromophenol blue] and heated for 5 min at 95°C prior to SDS-PAGE and immunoblotting.

### Preparation of 8-Oxoguanine DNA Substrate and *in vitro* BER Assay

A 5′-fluorescently labeled oligonucleotide containing 8-oxoguanine at position 20 (5′-IRDYE700-ATCTACCGAGTCC GTCCGAXCACGCTTATTGGCTACCGA-3′; where X is equivalent 8-oxoguanine) and the complementary oligo- nucleotide containing cytosine opposite the 8-oxoguanine lesion (5′-TCGGTAGCCAATAAGCGTG**C**TCGGACGGACTCG GTAGAT-3′; where bold C is equivalent to complementary cytosine) were annealed in TE buffer containing 200 mM NaCl by heating at 95°C for 5 min and slow cooling to room temperature to generate the 8-oxoguanine:C oligonucleotide duplex. Reactions (10 μl) contained 50 fmol DNA, 1 μg acetylated BSA in buffer containing 25 mM Tris–HCl (pH 8.0), 50 mM KCl, 2 mM ATP, 8.5 mM MgCl_2_, 0.5 mM EDTA, 8.5% glycerol, and 1 mM DTT, and which were incubated for 20 min at 37°C with agitation. Reactions were stopped by the addition of 10 μl formamide loading dye (95% formamide and 2.5 mg/ml bromophenol blue). Samples were heated for 5 min at 95°C prior to analysis by 10% denaturing PAGE (7 M urea) and substrate incision quantified using the Odyssey Image Analysis System.

### Alkaline Single Cell Gel Electrophoresis (Comet) Assay

The enzyme-modified comet assay was performed similar to that recently described (Carter et al., 2018, 2019), but under alkaline conditions. In brief and following cell lysis, slides were washed three times with enzyme reaction buffer (40 mM HEPES-KOH, 100 mM KCl, 0.5 mM EDTA and 0.2 mg/ml BSA, pH 8.0), and then incubated with either buffer alone (mock treated) or with buffer containing 5 pmol OGG1 and 0.6 pmol APE1 for 1 h at 37°C in a humidified chamber. Slides were transferred to an electrophoresis tank and incubated in the dark for 30 min in fresh cold electrophoresis buffer (300 mM NaOH, 1 mM EDTA, 1 % (v/v) DMSO, pH 13) to allow the DNA to unwind. Electrophoresis was performed at 25 V, 300 mA for 25 min, slides were neutralized with three 5 min washes of 0.5 M Tris–HCl (pH 8.0), and allowed to air dry overnight. Slides were rehydrated for 30 min in water (pH 8.0), stained for 30 min with SYBR Gold (Life Technologies, Paisley, United Kingdom) diluted 1:20,000 in water (pH 8.0) and allowed to air dry prior to imaging. Cells (50 per slide, 2 slides per time point) were analyzed using the Komet 6.0 image analysis software (Andor Technology, Belfast, United Kingdom). Percentage tail DNA values were averaged from at least three independent experiments.

## Results

### Purification of the Major E3 Ubiquitin Ligase Promoting OGG1 Ubiquitylation

**FIGURE 1:**
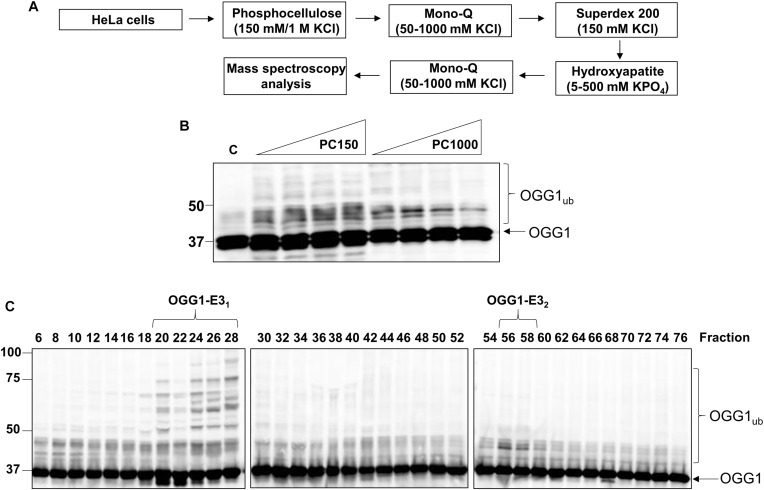
Purification of the major cellular E3 ubiquitin ligase for OGG1. **(A)** Scheme for the purification of the E3 ubiquitin ligase for OGG1 from HeLa cell extracts. **(B)** *In vitro* ubiquitylation of His-tagged OGG1 by low-salt (PC150) and high salt elution (PC1000) protein fractions generated from Phosphocellulose chromatography of HeLa whole cell extract. A control reaction **(C)** in the absence of any fraction is in the first lane, and increasing amounts (1, 2, 5, and 10 μg) of fraction were used. **(C)** *In vitro* ubiquitylation of His-tagged OGG1 using fractions from the first ion exchange (Mono Q) chromatography. Reactions were analyzed by SDS-PAGE and immunoblotting using OGG1 antibodies. Molecular weight markers are indicated on the left-hand side of the immunoblots, and the positions of unmodified and ubiquitylated OGG1 (OGG1_*ub*_) are displayed. Fractions containing E3 ubiquitin ligase activity for OGG1 (OGG1-E1_1_ and OGG1-E_2_) are indicated.

Given the vital role of OGG1 in prevention of mutagenesis and genome instability through excising 8-oxoguanine lesions in DNA, we hypothesized that cellular protein levels of OGG1 are tightly regulated by ubiquitylation. Therefore to identify the E3 ubiquitin ligase in human cells that promoted OGG1 ubiquitylation, we utilized our previously successful and unbiased approach (Parsons et al., 2008; Parsons et al., 2009; Edmonds et al., 2017; Williams and Parsons, 2018). This methodology involves the utilization of column chromatography to separate proteins present within HeLa cell extracts (Figure 1A), and then to examine the E3 ubiquitin ligase activity of protein fractions using OGG1 protein as a substrate, along with factors that promote ubiquitylation (one E1 activating enzyme, nine E2 conjugating enzymes and ubiquitin). From the first stage using Phosphocellulose chromatography, there was evidence of a monoubiquitylation activity targeting OGG1 present in the low salt elution fraction (PC150), as demonstrated by protein bands just below 50 kDa (equivalent to the size of 38.8 kDa OGG1 plus addition of 8 kDa ubiquitin) which were higher in intensity than the control reaction without any protein fraction (Figure 1B; compare lanes 1 and 2–5 and Supplementary Figure 1A). A much weaker ubiquitylation activity was present in the high salt elution fraction (PC1000) (Figure 1B; compare lanes 1 and 6–10). We subsequently focused on the activity present within PC150, which was then separated by ion exchange (Mono Q) chromatography (Supplementary Figure 2). This stage of the protein separation process revealed the presence of two E3 ubiquitin ligase activities targeting OGG1, with one promoting polyubiquitylation as revealed by multiple OGG1 protein band shifts (Figure 1C; OGG1-E3_1_, fractions 20–28 and Supplementary Figure 1B) and the other, much weaker activity, promoting OGG1 monoubiquitylation (OGG1-E3_2_, fractions 56–58).

**FIGURE 2:**
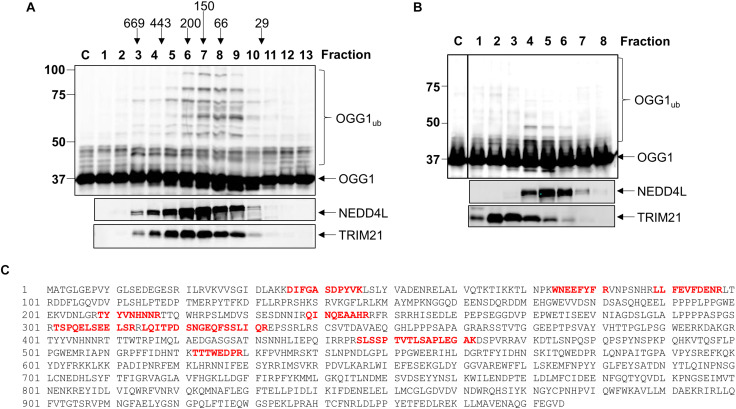
NEDD4L is an E3 ubiquitin ligase for OGG1 purified from human cell extracts. **(A)** *In vitro* ubiquitylation of His-tagged OGG1 by fractions generated from size exclusion (Superdex 200) chromatography. Above the figure are the positions of elution of known protein molecular weight standards. **(B)** *In vitro* ubiquitylation of His-tagged OGG1 by fractions obtained from the final ion exchange (Mono Q) chromatography. **(A,B)** contain immunoblotting of the appropriate fractions for the E3 ubiquitin ligases NEDD4L and TRIM21. **(C)** Protein sequence of NEDD4L with the peptides detected by mass spectrometry highlighted in red.

After the Mono Q chromatography stage, we again focused on the most significant ubiquitylation activity targeting OGG1 (OGG1-E3_1_), which was separated by size exclusion (Superdex 200) chromatography. The E3 ubiquitin ligase activity of these protein fractions demonstrated significant stimulation of OGG1 polyubiquitylation, but furthermore suggested that the activity corresponded to a protein or protein complex equivalent to a molecular weight of ∼66–200 kDa in size (Figure 2A; fractions 6–9 and Supplementary Figure 3A). Fractions containing this activity were subsequently separated by hydroxyapatite chromatography, and also by a final ion exchange (Mono Q) chromatography, and whilst the E3 ubiquitin ligase activity targeting OGG1 had reduced in intensity, this was still clearly visible (Figure 2B; fractions 4–6 and Supplementary Figure 3D). These purified fractions were subject to analysis by nanoLC-MS/MS tandem mass spectrometry which revealed the presence of proteins associated with protein ubiquitylation but more importantly three E3 ubiquitin ligases, NEDD4L, tripartite motif-containing protein 21 (TRIM21) and E3 ubiquitin-protein ligase NEDD4 (Table 1). NEDD4L, which displayed the highest Mascot score of these candidate E3 ubiquitin ligases, had a protein sequence coverage of 10% (Figure 2C). We therefore analyzed protein fractions purified from the size exclusion chromatography stage for the presence of NEDD4L and TRIM21, which both demonstrated a good alignment with OGG1 ubiquitylation activity (Figure 2A; lanes 5–9 and Supplementary Figures 3B,C). However, following the final ion exchange (Mono Q) chromatography, these proteins eluted differently and the E3 ubiquitin ligase activity targeting OGG1 demonstrated a more accurate alignment with the presence of NEDD4L (Figure 2B; lanes 4–7 and Supplementary Figures 3E,F).

**TABLE 1:** Mass spectrometry analysis of purified fractions containing OGG1-E3_1_.

Accession	Description	Mascot Score
P22314	Ubiquitin-like modifier activating enzyme 1	2510
P08107	Heat shock 70 kDa protein	1031
Q9Y3F4	Serine-threonine kinase receptor-associated protein	560
Q04323	UBX domain-containing protein 1	245
Q95155	Ubiquitin conjugation factor E4 B	390
Q94888	UBX domain-containing protein 7	326
Q9NZL4	Hsp70-binding protein 1	305
**Q96PU5**	**E3 ubiquitin-protein ligase NEDD4-like**	**200**
Q6PID6	Tetratricopeptide repeat protein 33	190
**P19474**	**E3 ubiquitin-protein ligase TRIM21**	**178**
Q9GZS3	WD repeat-containing protein 61	158
**P46934**	**E3 ubiquitin-protein ligase NEDD4**	**151**

*Candidate E3 ubiquitin ligases are highlighted in bold.*

### Characterization of NEDD4L as an E3 Ubiquitin Ligase for OGG1

**FIGURE 3:**
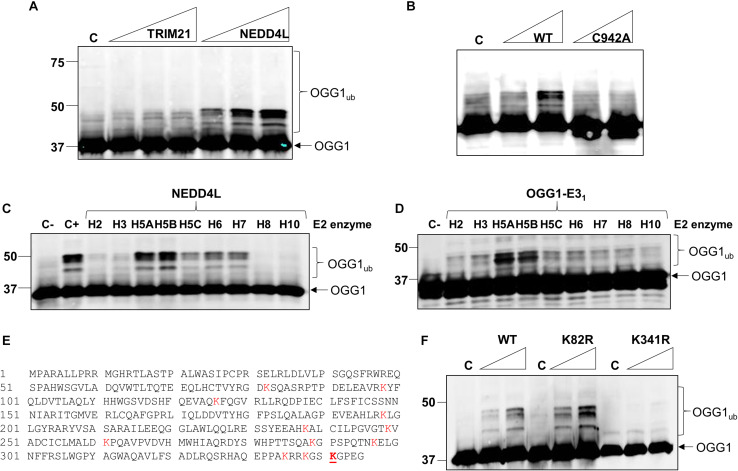
NEDD4L ubiquitylates OGG1 *in vitro* on lysine 341. **(A)** *In vitro* ubiquitylation of His-tagged OGG1 by His-tagged TRIM21 and His-tagged NEDD4L. Increasing amounts of TRIM21 (1.9, 3.7, and 7.4 pmol) and NEDD4L (1, 2.1 and 4.2 pmol) were used. **(B)** *In vitro* ubiquitylation of His-tagged OGG1 by His-tagged wild type and C942A NEDD4L (1 and 2.1 pmol). A control reaction **(C)** in the absence of any E3 ubiquitin ligase protein is in the first lane. *In vitro* ubiquitylation of OGG1 by **(C)** His-tagged NEDD4L (2 pmol) in the presence of individual E2 conjugating enzymes, or **(D)** an active fraction containing E3 ubiquitin ligase activity for OGG1 (OGG1-E3_1_) purified from HeLa whole cell extracts. Control reactions in the absence (C-) or presence (C+) of all E2 enzymes are in the first lane(s). **(E)** Protein sequence of OGG1 with the lysine residues highlighted in red, and lysine 341 the target for NEDD4L-ubiquitylation underlined in red. **(F)** *In vitro* ubiquitylation of His-tagged wild type (WT), K82R and K341R mutants of OGG1 by His-tagged NEDD4L. A control reaction **(C)** in the absence of NEDD4L is in lanes 1, 4 and 7, and increasing amounts of NEDD4L (1 and 2.1 pmol) were used. All *in vitro* ubiquitylation reactions were analyzed by SDS-PAGE and immunoblotting using OGG1 antibodies. Molecular weight markers are indicated on the left-hand side of *in vitro* ubiquitylation reactions and the positions of unmodified and ubiquitylated OGG1 (OGG1_*ub*_) are displayed.

Given that we had identified TRIM21 and NEDD4L as two candidate enzymes purified from HeLa cell extracts that have the potential to target OGG1 for ubiquitylation, in order to confirm which enzyme catalyzes this activity, we cloned *trim21* and *nedd4l* cDNA into a bacterial expression plasmid and then purified the recombinant proteins following overexpression. We were able to purify the full length recombinant E3 ubiquitin ligase proteins to a similar level of purity (Supplementary Figures 4A,B; see protein staining), although NEDD4L (112 kDa) was quite unstable and a certain degree of degradation was revealed by immunoblotting, in comparison to TRIM21 (54 kDa). Nevertheless, we examined the E3 ubiquitin ligase activity of both enzymes using OGG1 as a substrate, and demonstrated that only NEDD4L was capable of catalyzing OGG1 ubiquitylation (Figure 3A; lanes 5–7) and not TRIM21 (Figure 3A; lanes 1–4 and Supplementary Figure 5A). In addition, we created an enzymatically inactive form of NEDD4L protein with a mutation in the cysteine residue (C942A) present within the active site. Following overexpression and purification of recombinant NEDD4L-C942A from bacterial cells, we demonstrated that catalytically inactive NEDD4L-C942A was unable to ubiquitylate OGG1 *in vitro* when compared to wild-type NEDD4L (Figure 3B; compare lanes 2–3 to 4–5 and Supplementary Figure 5B). We consequently examined the specificity of NEDD4L for the E2 conjugation enzyme promoting OGG1 ubiquitylation, and show that it displays preference for the H5a and H5b E2 enzymes, and to a lesser extent H5c, H6, and H7 (Figure 3C and Supplementary Figure 5C). This E2 enzyme specificity was similar to that shown by the E3 ubiquitin ligase activity purified from HeLa cell extracts (Figure 3D and Supplementary Figure 5D), suggesting that the enzyme is most likely NEDD4L. In an attempt to identify the site of OGG1 ubiquitylation by NEDD4L, we generated a series of single point mutations at the 12 lysine residues present within OGG1 (Figure 3E). Following overexpression and purification of the proteins from bacterial cells, we subsequently discovered that mutation of lysine 341 to arginine (K341R) at the C-terminal end of the OGG1 protein completely suppressed ubiquitylation by NEDD4L *in vitro* (Figure 3F; compare lanes 2–3 and 8–9 and Supplementary Figure 5E), suggesting that this lysine residue is the specific target for NEDD4L-dependent ubiquitylation. In comparison, and as an example, mutation of lysine 82 to arginine at the N-terminal end of the OGG1 protein did not inhibit ubiquitylation by NEDD4L (Figure 3F; compare lanes 2–3 and 5–6).

### Cellular Steady-State Protein Levels of OGG1 Are Predominantly Unaffected by Absence of NEDD4L

**FIGURE 4:**
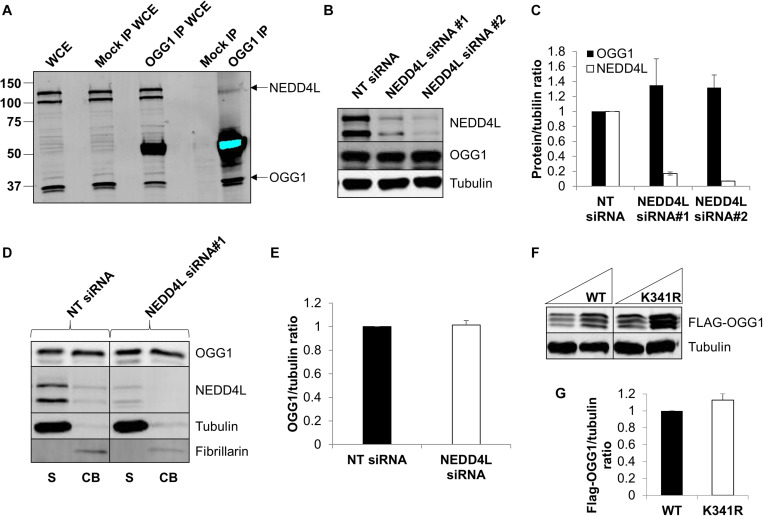
NEDD4L interacts with cellular OGG1 but does not control steady-state OGG1 protein levels. **(A)** Interaction of NEDD4L with OGG1 in U2OS cells following incubation of whole cell extracts (WCE) with OGG1 antibodies (OGG1 IP) or with magnetic beads only (Mock IP). WCE (20 μg) and proteins bound to the beads were analyzed by SDS-PAGE and immunoblotting with OGG1 and NEDD4L antibodies. **(B–E)** Analysis of OGG1 protein stability in the presence of non-targeting (NT) control siRNA or NEDD4L siRNA (siRNA#1 and siRNA#2) for 72 h. **(B)** WCE was analyzed by SDS-PAGE and immunoblotting. **(C)** Protein levels of OGG1 and NEDD4L relative to tubulin (mean ± SE) were quantified and normalized relative to the NT siRNA transfected cells which was set to 1.0. **(D)** Soluble (S) and chromatin bound (CB) fractions were analyzed by SDS-PAGE and immunoblotting. **(E)** Protein levels of OGG1 relative to tubulin (mean ± SD) in the soluble fraction were quantified and normalized relative to the NT siRNA transfected cells which was set to 1.0. **(F)** Analysis of the stability of wild type (WT) and OGG1 mutant (K341R) proteins in U2OS cells by SDS-PAGE and immunoblotting of WCE. **(G)** Levels of Flag-tagged WT and K341R OGG1 proteins relative to tubulin (mean ± SD) were quantified and normalized relative to the WT-OGG1 transfected cells which was set to 1.0. All data was acquired from at least three independent experiments.

To confirm that OGG1 and NEDD4L interact in human cells, we immunoprecipitated endogenous OGG1 from U2OS whole cell extracts. Indeed, we were able to show that a small proportion of NEDD4L can be found in association with OGG1, compared to the mock immunoprecipitation, even in the absence of any exogenous stress (Figure 4A; compare lanes 4 and 5 and Supplementary Figure 6A). We subsequently analyzed the cellular steady-state protein levels of OGG1 following depletion of *nedd4l* using two individual siRNA sequences that were effective (>85%) in suppressing the protein levels of NEDD4L versus the non-targeting (NT) control siRNA (Figures 4B,C and Supplementary Figures 6B–D). This revealed that OGG1 protein levels increased by 35 and 32%, respectively although the data was not statistically significant. We therefore separated cellular proteins by biochemical fractionation into soluble and chromatin bound fractions. This demonstrated that the majority of NEDD4L protein was present in the soluble fraction and not associated with chromatin, whereas OGG1 was equally distributed between both fractions (Figure 4D; compare lanes 1 and 2 and Supplementary Figures 6E–H). Following NEDD4L siRNA, OGG1 stability in the soluble, NEDD4L-containing fraction did not increase in comparison to the NT control siRNA (Figure 4D; compare lanes 1 and 3 and Figure 4E). No significant changes in the levels of OGG1 in the chromatin bound fractions were also observed (Figure 4D; compare lanes 2 and 4), demonstrating that NEDD4L has no dramatic impact on the steady state regulation of OGG1 protein levels. Since we determined that lysine 341 is the major ubiquitylation site within OGG1 catalyzed by NEDD4L *in vitro* (Figure 3F), we also examined the comparative stability of wild type and K341R OGG1 proteins following expression in U2OS cells (Figure 4F and Supplementary Figures 6I,J). We found that there was only a modest (∼15%) increase in stability of the K341R mutant versus the wild type protein, which was not statistically significant (Figure 4G). Note that a triplet of bands was observed for both expressed OGG1 proteins due to sequential loss of the 3xFLAG-tags (e.g., during expression, purification or SDS-PAGE analysis), but that these displayed no differences in intensity or stability, suggesting that ubiquitylation of OGG1 at lysine 341 has no dramatic impact on stability of newly synthesized OGG1 protein.

### DNA Damage-Inducible Protein Levels of OGG1 Are Modulated by NEDD4L, Which Also Controls OGG1 Activity

**FIGURE 5:**
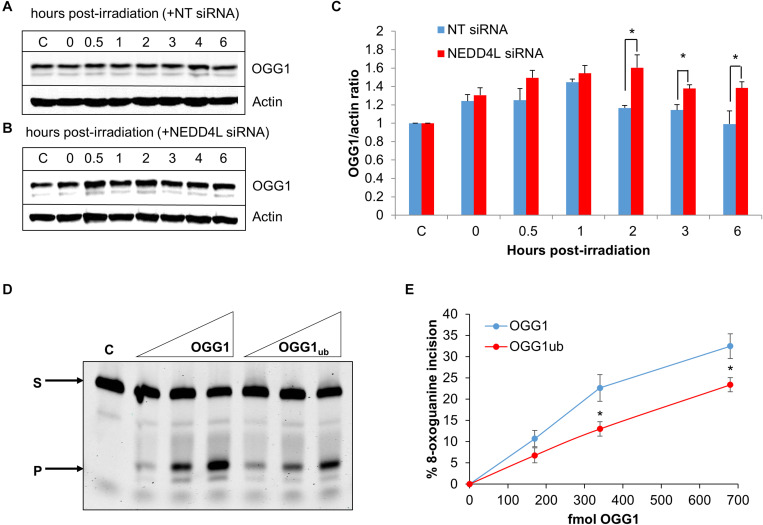
NEDD4L controls OGG1 protein levels in response to DNA damage. **(A–C)** U2OS cells treated with **(A)** non-targeting (NT) control siRNA or (B) NEDD4L siRNA, were either unirradiated **(C)** or treated with x-ray irradiation (10 Gy) and harvested at the indicated time points post-treatment. Whole cell extracts were prepared and analyzed by SDS-PAGE and immunoblotting. **(C)** Protein levels of OGG1 protein relative to actin (mean ± SD) were quantified from at least three independent experiments, and were normalized relative to the respective unirradiated cells which was set to 1.0. ^∗^*p* < 0.05 as analyzed by a two sample *t*-test. **(D–E)** Incision of 8-oxoguanine duplex oligonucleotide substrate in the presence of increasing amounts of OGG1 or ubiquitylated OGG1 (170, 340, or 680 fmol). *In vitro* ubiquitylation of OGG1 was performed prior to the incision assay in the presence of either His-tagged NEDD4L or heat-denatured His-tagged NEDD4L (4.2 pmol). **(E)** Shown is the mean percentage substrate incision ± SD from at least three independent experiments. ^∗^*p* < 0.05, as analyzed by a one sample *t*-test.

To analyze dependence of NEDD4L on regulating the cellular levels of OGG1 in response to oxidative DNA damage, we treated U2OS cells with x-ray irradiation and monitored stability of OGG1 at several time points post-IR in the absence versus the presence of NEDD4L siRNA. In cells treated with NT control siRNA, the stability of OGG1 increased immediately post-IR and was maximal at 1 h post-IR where the levels were ∼1.45-fold higher than unirradiated cells (Figures 5A,C and Supplementary Figures 7A,B). In contrast in the presence of NEDD4L siRNA, the stability of OGG1 was prolonged from 2 to 6 h post-IR where they were statistically significantly higher than NT control siRNA cells (Figures 5B,C and Supplementary Figures 7C,D). To also investigate whether NEDD4L had any impact on OGG1 DNA glycosylase activity, we examined excision of a fluorescently labeled 8-oxoguanine containing duplex oligonucleotide using NEDD4L-ubiquitylated OGG1 protein prepared *in vitro*, compared to non-ubiquitylated OGG1 protein. We found that the 8-oxoguanine substrate was 1.4–1.7-fold less efficiently cleaved by ubiquitylated OGG1 compared to non-ubiquitylated OGG1 (Figure 5D; compare lanes 2–4 and 5–7 and Figure 5E). This difference in activity is observable even though only ∼20 % of the OGG1 can be ubiquitylated by NEDD4L *in vitro* (Figure 3A), but which nevertheless demonstrates that NEDD4L-dependent ubiquitylation of OGG1 controls its DNA glycosylase/lyase activity.

### NEDD4L Regulates DNA Damage Repair and Cell Survival in Response to IR

**FIGURE 6:**
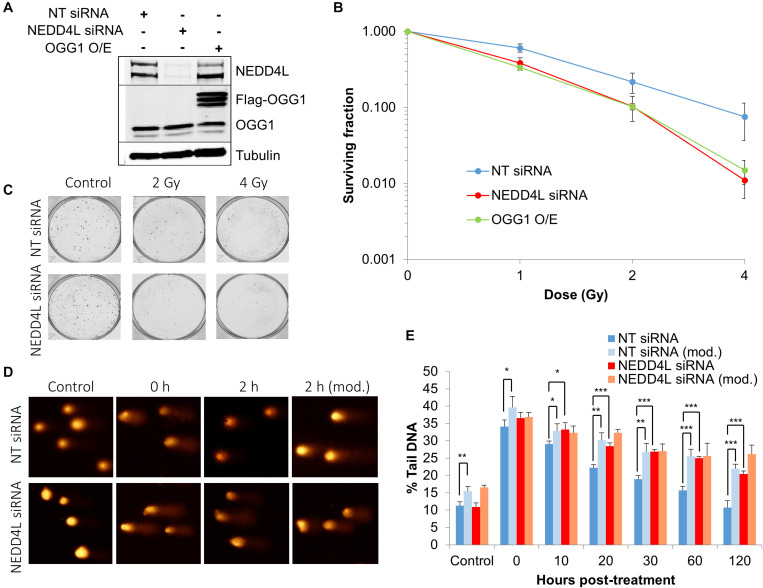
NEDD4L is required to control DNA damage repair and cell survival in response to IR. **(A)** WCE from U2OS cells treated with NT control siRNA, NEDD4L siRNA, or following OGG1 overexpression were prepared and analyzed by SDS-PAGE and immunoblotting. **(B–C)** Clonogenic survival of cells was analyzed following treatment with increasing doses of x-ray irradiation (0-4 Gy). Shown is the mean surviving fraction with standard errors from at least three independent experiments, as well as respective images from the colonies formed. **(D,E)** Cells were irradiated with x-ray irradiation (1.5 Gy) and DNA damage measured at various time points post-IR by the enzyme modified alkaline comet assay following incubation in the absence or presence (revealing residual oxidative DNA damage; as indicated by mod) of the recombinant enzymes APE1 and OGG1. Shown is the mean % tail DNA ± SD. *p < 0.05, **p < 0.005, ***p < 0.001 as analyzed by a one sample *t*-test. Also shown are respective images of the comets formed.

In order to examine the impact of the enhanced lifetime of OGG1 post-IR in the absence of NEDD4L, but also the increased activity of OGG1 under these conditions, we analyzed overall cell survival in cells treated with NEDD4L siRNA. Additionally, we included cells containing OGG1 overexpression (Figure 6A and Supplementary Figures 8A–C), which would mimic the impact of NEDD4L loss. We demonstrated that NEDD4L depleted cells displayed significantly (*p* < 0.001) reduced cell survival in response to x-ray irradiation compared to the NT control siRNA treated cells (Figures 6B,C). Furthermore, this phenotype could be reproduced following overexpression of OGG1 (*p* < 0.005 compared to NT siRNA treated cells), demonstrating that increased levels and thus activity of OGG1 directly enhances the cell killing effects of IR (Figure 6B). We subsequently analyzed the impact of this increased stability of OGG1 post-IR, on the kinetics of DNA damage repair in cells treated with NEDD4L siRNA. Alkaline comet assays were used to measure the kinetics of DNA single strand breaks and alkali-labile sites generated directly but also as intermediates of BER. Post-IR, we observed that DNA damage was gradually resolved in NT control siRNA treated cells from 0 to 120 min post-irradiation (Figures 6D,E, dark blue bars). In contrast in NEDD4L siRNA treated cells, the levels of DNA damage were persistently higher, and were statistically significant at 10–120 min post-irradiation than the NT control siRNA treated cells (Figures 6D,E, compare dark blue and red bars). We hypothesized that this increased DNA damage in NEDD4L depleted cells was caused through increased OGG1-mediated BER. Therefore, we utilized a modified version of the comet assay, employing recombinant OGG1 and APE1 (to increase OGG1 processivity) to incise any residual oxidative DNA damage at the various time-points post-irradiation. This revealed elevated levels of DNA damage in NT control siRNA treated cells from 20 to 120 min post-IR compared with the same cells analyzed under the standard alkaline comet assay conditions (Figures 6D,E, compare dark blue and light blue bars). This demonstrated the presence of unrepaired oxidative DNA damage during these time points, which have slower kinetics of repair than directly induced DNA single strand breaks and alkali-labile sites. However, in NEDD4L siRNA treated cells, there was no impact of the enzyme-modified conditions on the levels of DNA damage (Figures 6D,E, compare red and orange bars). This suggests that oxidative DNA damage sites have been more readily incised by the increased levels and activity of OGG1 in the absence of NEDD4L, resulting in an overall elevation in BER intermediates which likely contribute to decreased cell survival post-IR.

## Discussion

The BER pathway is the major DNA repair pathway which co-ordinates the repair of oxidative DNA base damage and therefore prevents the build-up of premutagenic lesions that can threaten genome stability. OGG1 plays a specific role in the repair of 8-oxoguanine lesions that are well known and established to promote GC to TA transversions in DNA. Whilst mice deficient in OGG1 only display moderate increases in spontaneous mutation rates associated with accumulation of 8-oxoguanine (Klungland et al., 1999; Minowa et al., 2000; Sakumi et al., 2003), altered OGG1 protein expression and activity has been observed in a number of different cancer cell types, including prostate, head and neck squamous cell carcinoma and colorectal cancer (Kondo et al., 2000; Trzeciak et al., 2004; Paz-Elizur et al., 2006; Kumar et al., 2012). Furthermore, a well-characterized single nucleotide polymorphism of OGG1 (serine 326 to cysteine) exists that lacks efficient repair of 8-oxoguanine and is associated with a higher risk of cancer development (Karahalil et al., 2012). In this study, and utilizing an unbiased approach for identifying E3 ubiquitin ligase activities present within human cell extracts, we have now identified NEDD4L that targets OGG1 for ubiquitylation on lysine 341 *in vitro* and which reduces its DNA glycosylase/lyase activity. Furthermore, we have shown that whilst depletion of NEDD4L using siRNA had no impact on the cellular steady-state protein levels of OGG1 protein, in response to oxidative DNA damage caused by x-ray irradiation OGG1 displayed a prolonged stability. As a consequence of this increased stability and incision activity of OGG1 in the absence of NEDD4L, we demonstrate that this caused enhanced DNA damage repair capacity but led to formation of BER intermediates that contributed to an increase in IR-induced cell killing.

NEDD4L is one of the nine members of the NEDD4 family of E3 ubiquitin ligases, which share a similar structural architecture consisting of an N-terminal calcium and phospholipid binding domain, two-four central WW domains for substrate recognition, and a C-terminal HECT domain for catalytic activity (Escobedo et al., 2014; Sluimer and Distel, 2018). NEDD4L was originally identified as an E3 ubiquitin ligase that ubiquitylates and degrades epithelial sodium channels in the regulation of hypertension (Rizzo and Staub, 2015). However, NEDD4L has also been recognized to target other proteins including transforming growth factor β (TGF-β), Smad2/3 and Dishevelled2 (Goel et al., 2015), which are vital in cell signaling processes required for normal cell physiology. This is reflected in the multiple pathologies that are observed in NEDD4L knockout mice (Manning and Kumar, 2018). We now add to this evidence by demonstrating that purified and recombinant NEDD4L can target OGG1 and regulate its DNA glycosylase/lyase activity *in vitro*, and controls OGG1 protein levels in cultured cells particularly in response to DNA damage stress. Interestingly, the NEDD4 family has previously been demonstrated to play a role in the DNA damage response, particularly by controlling the ubiquitylation and nuclear localization of BRCA1-associated ATM Activator 1 (BRAT1) that maintains ATM phosphorylation status (Low et al., 2015). Also DNA damage binding protein 2 (DDB2), which is well known to play a role in nucleotide excision repair but also acts as a transcriptional regulator, has been shown to downregulate NEDD4L expression (Zhao et al., 2015). Given these major roles of NEDD4L in cell signaling and the cellular DNA damage response, it is not surprising that absence of NEDD4L is associated with disease development, particularly cancer. Indeed, NEDD4L is significantly downregulated at the gene and protein level in colorectal cancer (Eide et al., 2013; Tanksley et al., 2013), non-small cell lung cancer and breast cancer (Guarnieri et al., 2018) amongst others, and is associated with a poor prognosis. Therefore, NEDD4L has been identified as having a tumor suppressor role. An interesting question is whether there is a backup E3 ubiquitin ligase for OGG1 in cells, which may compensate for absence of NEDD4L or mutations causing loss of function. Our results demonstrated that there are additional activities for OGG1 present in fractionated extracts (e.g., OGG1-E1_2_ promoting monoubiquitylation) that may perform such a role. However, these E3 ubiquitin ligase enzymes need to be identified and fully characterized in terms of their impact on OGG1 activity and protein levels, as well as the site of ubiquitylation relative to NEDD4L.

It is noteworthy that we observed that NEDD4L is predominantly present within the soluble and cytosolic cell compartment upon biochemical fractionation, and that the enzyme most likely regulates the OGG1 protein present within this fraction in response to oxidative stress, and not the protein bound to chromatin. This is consistent with our other previous studies, including those on Pol β and PNKP (Parsons et al., 2008; Parsons et al., 2009; Parsons et al., 2012; Carter and Parsons, 2016), demonstrating that ubiquitylation-dependent regulation of BER protein levels occurs within the cytosol and that this is a mechanism for allowing increases in protein that can then be transported to the nucleus to promote DNA damage repair. We found that the elevation in OGG1 protein levels are relatively moderate post-IR (up to 1.45-fold), although again this is consistent with our previously reported evidence and which shows that BER is finely tuned according to the levels of DNA damage stress. However, predictably there should exist another E3 ubiquitin ligase(s) that specifically regulates the nuclear protein levels of OGG1 and/or its association with chromatin. Indeed, in the initial chromatography fractionation of HeLa cell extracts we demonstrated that another, albeit weaker, ubiquitylation activity exists in the PC1000 fraction which largely contains proteins that have an affinity for DNA. This enzymatic activity needs to be purified and identified for subsequent detailed analysis. Another question that needs to be resolved is the sensing mechanism that modulates the E3 ubiquitin ligase activity of NEDD4L towards OGG1 in response to oxidative stress. This could occur either at the transcriptional level, and speculatively may involve DDB2 as described above, or could involve regulation of enzymatic activity through post-translational modifications. Indeed, the C-terminus of OGG1 containing the lysine 341 ubiquitylation site discovered here, has been shown to be subjected to a number of different post-translational modifications, including phosphorylation and acetylation (Carter and Parsons, 2016). Interestingly, lysine 341 and additionally lysine 338, have been shown to be targets for acetylation by the acetyltransferase, p300 (Bhakat et al., 2006). It was discovered that following oxidative stress, the level of acetylated OGG1 increased and enhanced enzymatic turnover. It is therefore tempting to suggest a model that NEDD4L and p300 act as molecular switches to either inhibit or activate, respectively the activity of OGG1 in response to DNA damage by targeting lysine 341. This could be a mechanism through which the BER pathway efficiently responds to cellular oxidative DNA damage and helps promote genome stability. The potential cross-talk between ubiquitylation and acetylation (and other PTMs) of OGG1 though, requires further investigation.

Interestingly, we demonstrated that prolonged stability of OGG1 in response to DNA damage in cells treated with NEDD4L siRNA led to enhanced capacity for BER, as demonstrated by increases in DNA repair intermediates post-IR, but which lead to elevated cell killing in response to IR. We also demonstrated that NEDD4L regulates the efficiency of OGG1 DNA glycosylase/lyase activity in addition to protein stability, which most likely contributes to the observed elevations in BER intermediates. Our data correlates with previous observations in TK6 lymphoblast cells demonstrating that overexpression of OGG1 enhanced IR-induced cell death (Yang et al., 2004; Yang et al., 2006). This suggests that the balance of the levels and activity of the protein are critical for controlling cell survival post-IR by preventing the formation of toxic DNA repair intermediates, such as DNA single strand breaks that can potentially lead to DNA double strand breaks following DNA replication. One of these studies further demonstrated that overexpression of OGG1 conversely led to increased resistance to hydrogen peroxide-induced DNA damage, and suggested that the differences in these phenotypes were caused by increased processing of complex/clustered DNA damage sites generated by IR that led to elevated formation of lethal DNA double strand breaks (Yang et al., 2006). Whilst this is an interesting hypothesis which requires validation, it should be noted that the frequency of these complex DNA lesions are likely to be very low using x-ray and γ-irradiation due to their low ionization density. Nevertheless, it will be important going forward to comparatively analyze the impact of NEDD4L depletion on the cellular response to hydrogen peroxide-induced DNA damage compared to those obtained herein using x-ray irradiation utilizing multiple cell line model systems, and to specifically monitor DNA damage repair kinetics and the links with cell survival.

## Data Availability Statement

The raw data supporting the conclusions of this article will be made available by the authors, without undue reservation.

## Author Contributions

JP conceptualized and designed the project. JH performed the experiments and helped to draft the manuscript. Both authors performed data analysis and validation and reviewed and edited the manuscript.

## Conflict of Interest

The authors declare that the research was conducted in the absence of any commercial or financial relationships that could be construed as a potential conflict of interest.
