# Supplementary material for: The E3 Ubiquitin Ligase NEDD4L Targets OGG1 for Ubiquitylation and Modulates the Cellular DNA Damage Response
Source: Front Cell Dev Biol. 2020 Nov 12;8:607060. doi: 10.3389/fcell.2020.607060 (PMC7688902; doi:10.3389/fcell.2020.607060)
Supplement: Supplementary file 1 [file Table_1.DOCX]

Supplementary Material


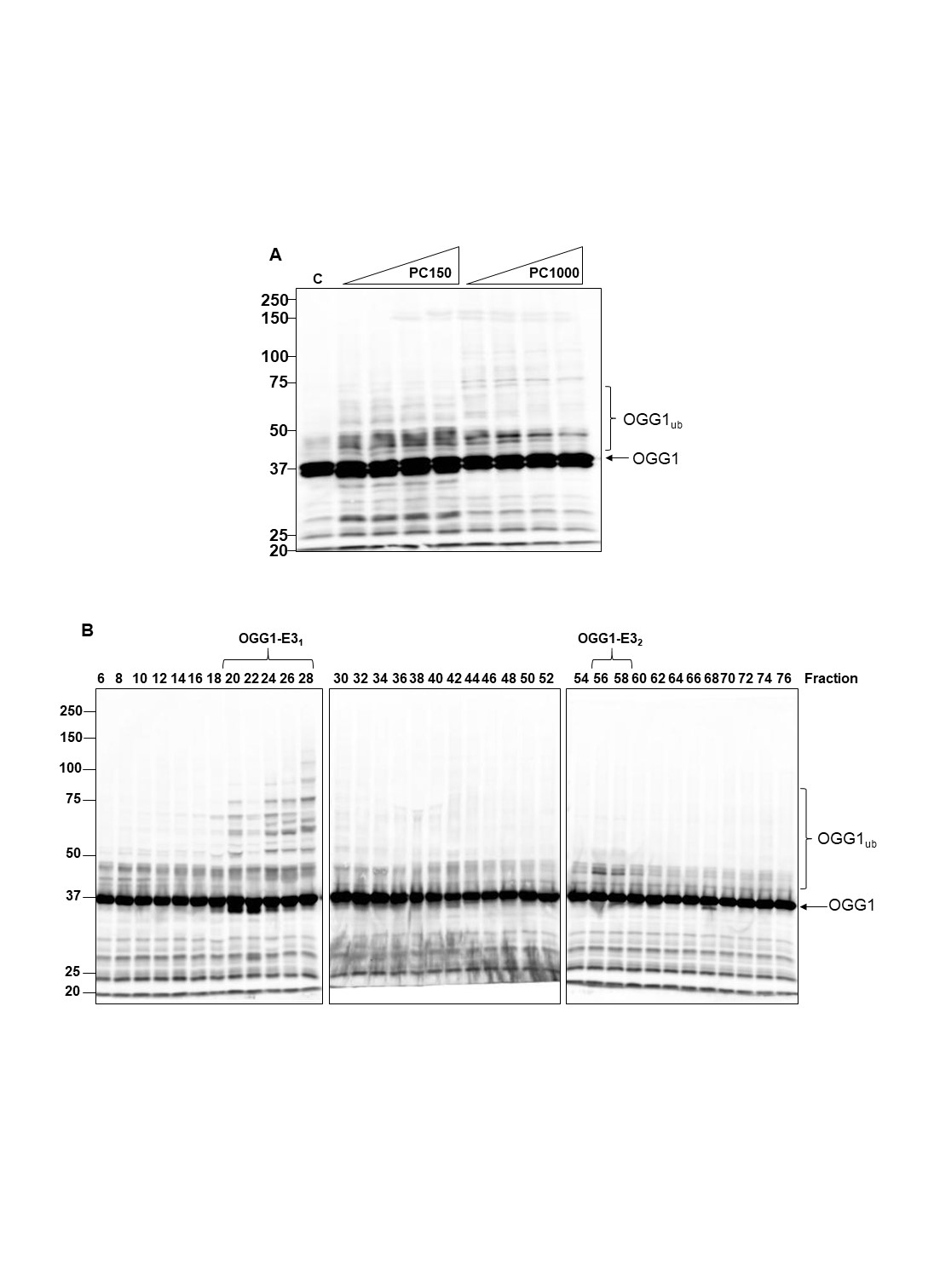


**Supplementary Figure 1.** Purification of the major cellular E3 ubiquitin ligase for OGG1. (**A**) *In vitro* ubiquitylation of His-tagged OGG1 by low-salt (PC150) and high salt elution (PC1000) Phosphocellulose protein fractions. A control reaction (C) in the absence of any fraction is in the first lane, and increasing amounts (1, 2, 5 and 10 µg) of fraction were used. (**B**) *In vitro* ubiquitylation of His-tagged OGG1 using fractions from the first ion exchange (Mono Q) chromatography. Reactions were analyzed by SDS-PAGE and immunoblotting using OGG1 antibodies. Molecular weight markers are indicated on the left-hand side of the immunoblots, and the positions of unmodified and ubiquitylated OGG1 (OGG1_ub_) are displayed. Fractions containing E3 ubiquitin ligase activity for OGG1 (OGG-E1_1_ and OGG1-E_2_) are indicated. Full length blots are shown.


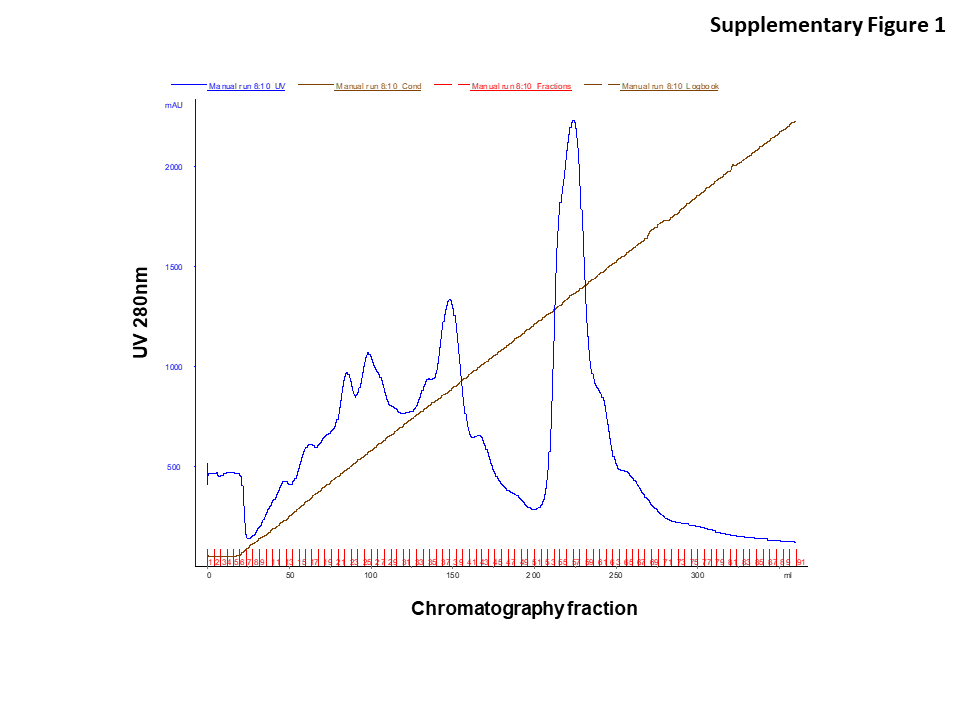


**Supplementary Figure 2.** Protein elution profile of Mono Q chromatography fractionation of HeLa PC150 fraction. Shown is the level of protein by UV detection (280 nm) following a salt gradient elution (50-1000 mM) of proteins from the PC150 fraction bound to the Mono Q chromatography column.


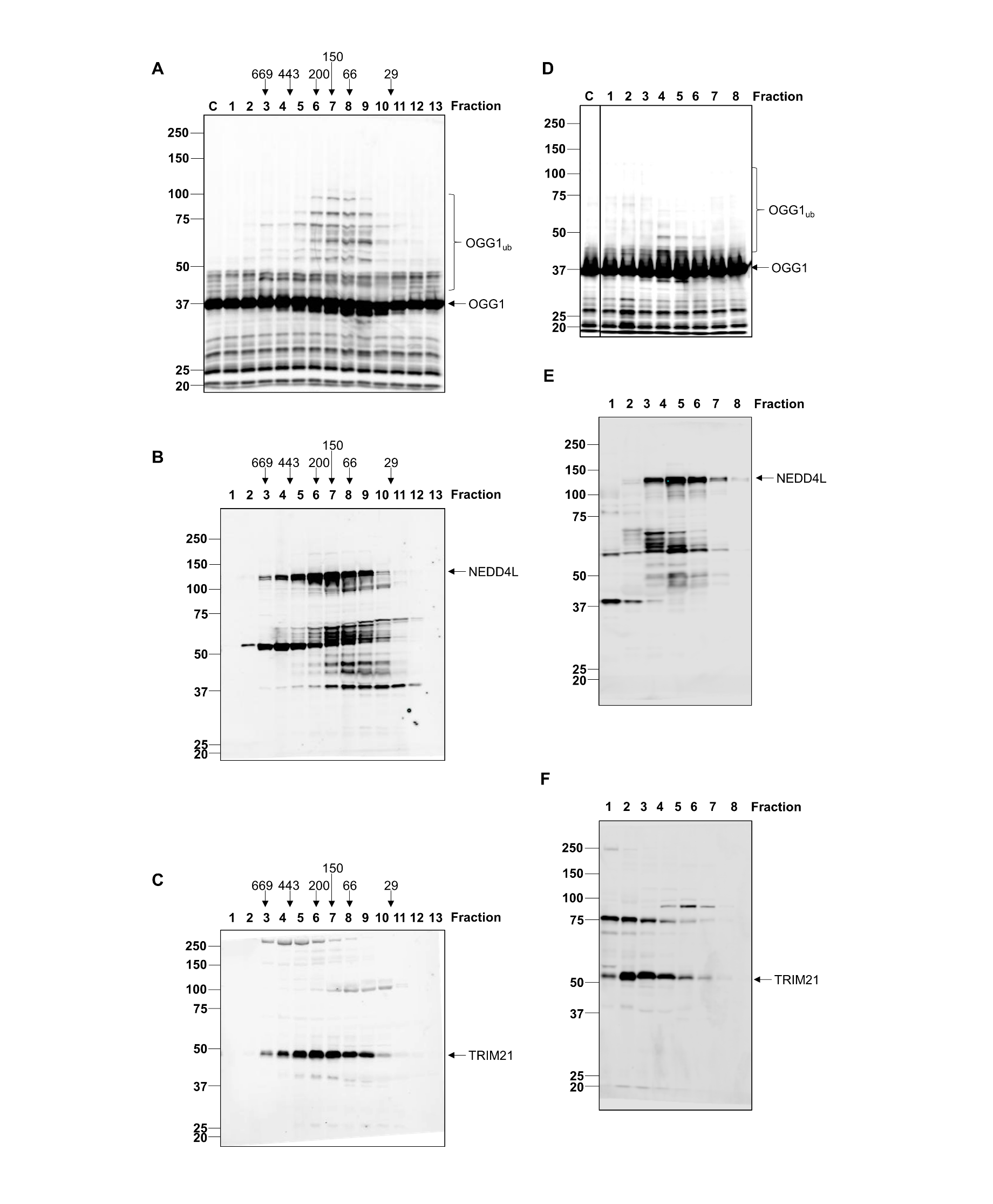


**Supplementary Figure 3.** NEDD4L is an E3 ubiquitin ligase for OGG1 purified from human cell extracts. (**A**) *In vitro* ubiquitylation of His-tagged OGG1 by fractions generated from size exclusion (Superdex 200) chromatography. Above the figure are the positions of elution of known protein molecular weight standards. Fractions were also analyzed by immunoblotting for the presence of the E3 ubiquitin ligases (**B**) NEDD4L and (**C**) TRIM21. (**D**) *In vitro* ubiquitylation of His-tagged OGG1 by fractions obtained from the final ion exchange (Mono Q) chromatography. The immunoblot was probed with antibodies specific for OGG1. Fractions were also analyzed by immunoblotting for the presence of the E3 ubiquitin ligases (**E**) NEDD4L and (**F**) TRIM21. NEDD4L displays some degree of degradation during sample processing/analysis. Molecular weight markers are indicated on the left-hand side of the immunoblots, and the positions of unmodified and ubiquitylated OGG1 (OGG1_ub_) are displayed in the appropriate figures. Full length blots are shown.


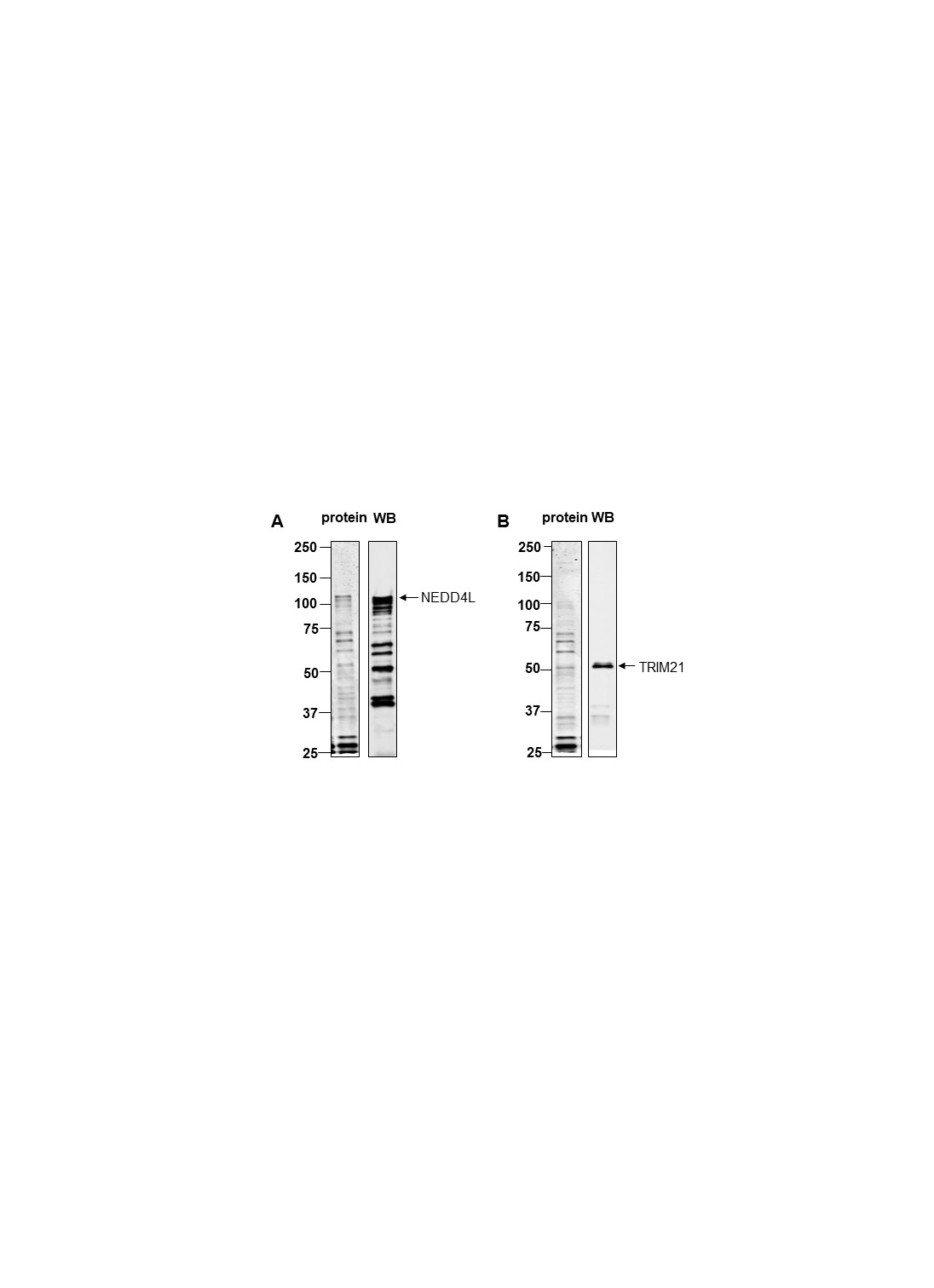


**Supplementary Figure 4.** Purity of recombinant His-tagged NEDD4L and TRIM21 purified from bacterial overexpression. Analysis of (**A**) His-tagged NEDD4L and (**B**) TRIM21 by SDS-PAGE and subsequent protein staining (left panels) or immunoblotting (right panels). Molecular weight markers are indicated on the left-hand side of the immunoblots.


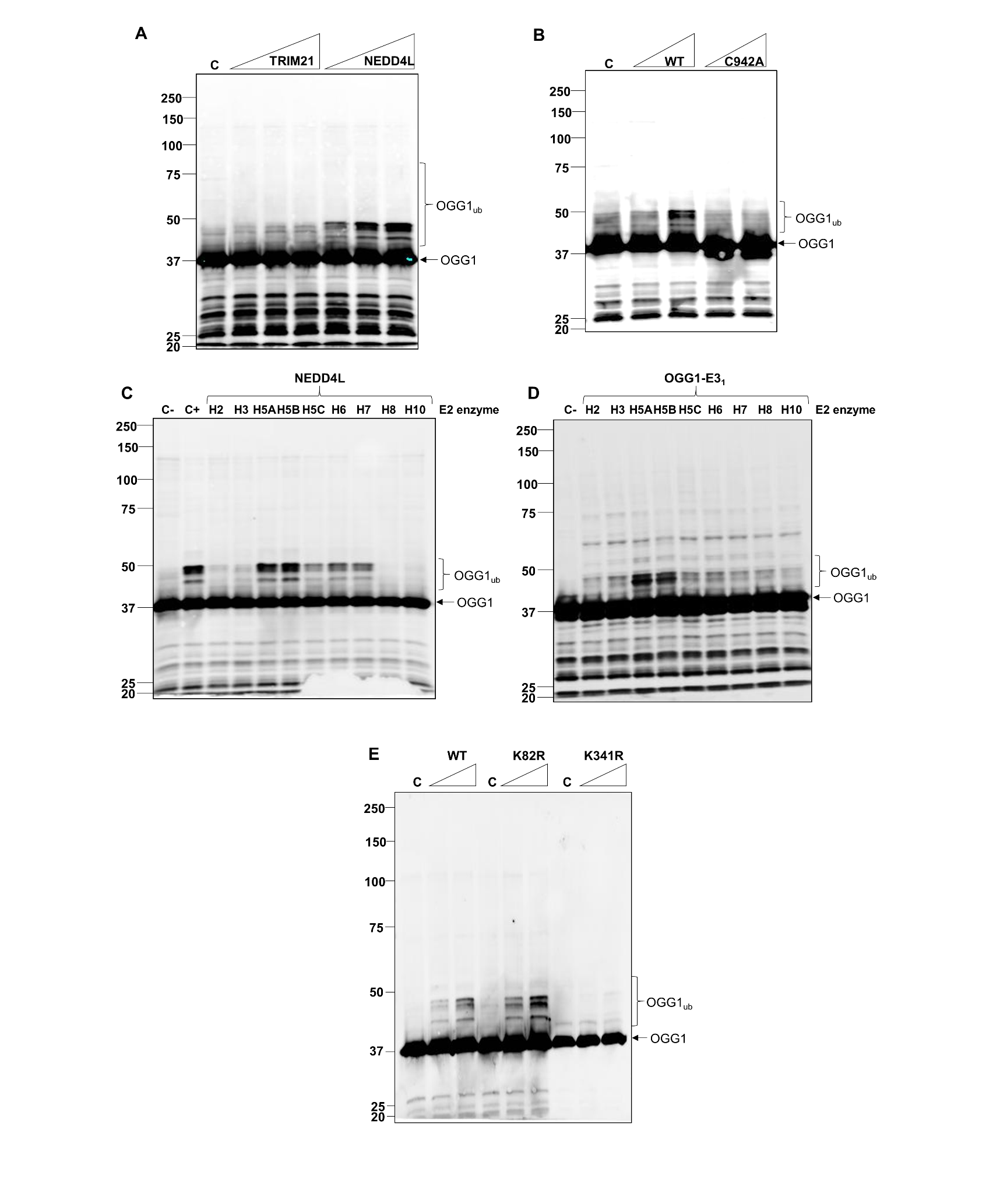


**Supplementary Figure 5.** NEDD4L ubiquitylates OGG1 *in vitro* on lysine 341. (**A**) *In vitro* ubiquitylation of His-tagged OGG1 by His-tagged TRIM21 and His-tagged NEDD4L. Increasing amounts of TRIM21 (1.9, 3.7 and 7.4 pmol) and NEDD4L (1, 2.1 and 4.2 pmol) were used. (**B**) *In vitro* ubiquitylation of His-tagged OGG1 by His-tagged wild type and C942A NEDD4L (1 and 2.1 pmol). A control reaction (C) in the absence of any E3 ubiquitin ligase protein is in the first lane of the appropriate blots. *In vitro* ubiquitylation of OGG1 by (**C**) His-tagged NEDD4L (2 pmol) or (**D**) an active fraction containing E3 ubiquitin ligase activity for OGG1 (OGG1-E3_1_) purified from HeLa whole cell extracts, in the presence of individual E2 conjugating enzymes. Control reactions in the absence (C-) or presence (C+) of all E2 enzymes are in the first lanes of the appropriate blots. (**E**) *In vitro* ubiquitylation of His-tagged wild type (WT), K82R and K341R mutants of OGG1 by His-tagged NEDD4L. A control reaction (C) containing the appropriate OGG1 proteins but in the absence of NEDD4L is in lanes 1, 4 and 7, and increasing amounts of NEDD4L (1 and 2.1 pmol) were used (lanes 2-3, 5-6 and 8-9). All *in vitro* ubiquitylation reactions were analyzed by SDS-PAGE and immunoblotting using OGG1 antibodies. Molecular weight markers are indicated on the left-hand side of the immunoblots. and the positions of unmodified and ubiquitylated OGG1 (OGG1_ub_) are displayed. Full length blots are shown.


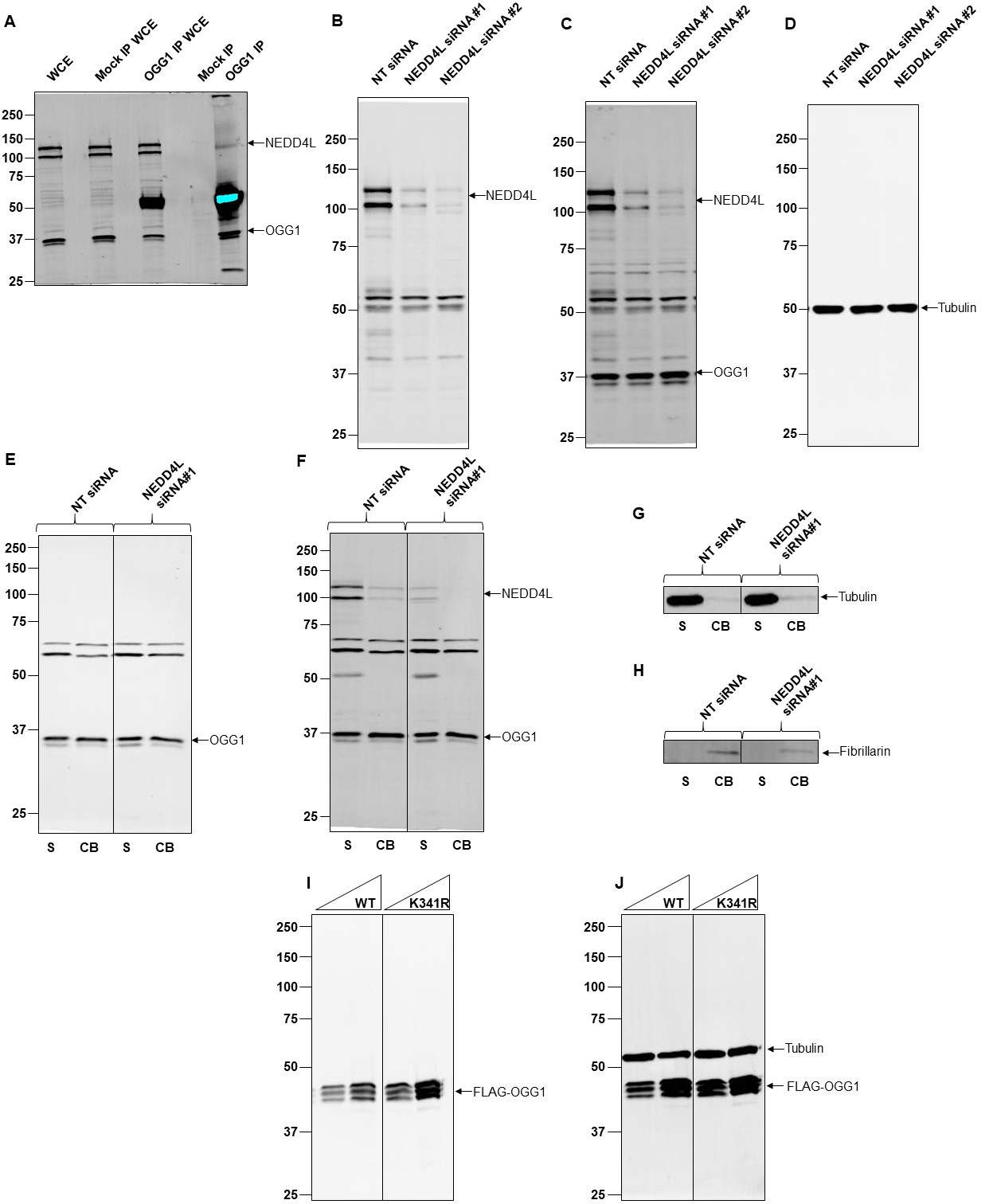


**Supplementary Figure 6.** NEDD4L interacts with cellular OGG1 but does not control steady-state OGG1 protein levels. (**A**) Interaction of NEDD4L with OGG1 in U2OS cells following incubation of whole cell extracts (WCE) with OGG1 antibodies (OGG1 IP) or with magnetic beads only (Mock IP). WCE (20 µg) and proteins bound to the beads were analyzed by SDS-PAGE and immunoblotting with OGG1 and NEDD4L antibodies. (**B-H**) Analysis of OGG1 protein stability in the presence of non-targeting (NT) control siRNA or NEDD4L siRNA (siRNA#1 and siRNA#2) for 72 h. (**B-D**) WCE was analyzed by SDS-PAGE and immunoblotting using (**B**) NEDD4L antibodies, followed by (**C**) OGG1 and (**D**) tubulin antibodies. (**E-H**) Soluble (S) and chromatin bound (CB) fractions were analyzed by SDS-PAGE and immunoblotting using (**E**) OGG1 followed by (**F**) NEDD4L antibodies, or (**G**) tubulin and (**H**) fibrillarin antibodies. (**I-J**) Analysis of the stability of wild type (WT) and OGG1 mutant (K341R) proteins in U2OS cells by SDS-PAGE and immunoblotting of WCE using (**I**) FLAG-tag antibodies followed by (**J**) tubulin antibodies. Full length blots are shown, except for (**G**) and (**H**) as membranes were cut to the size of the target proteins.

**
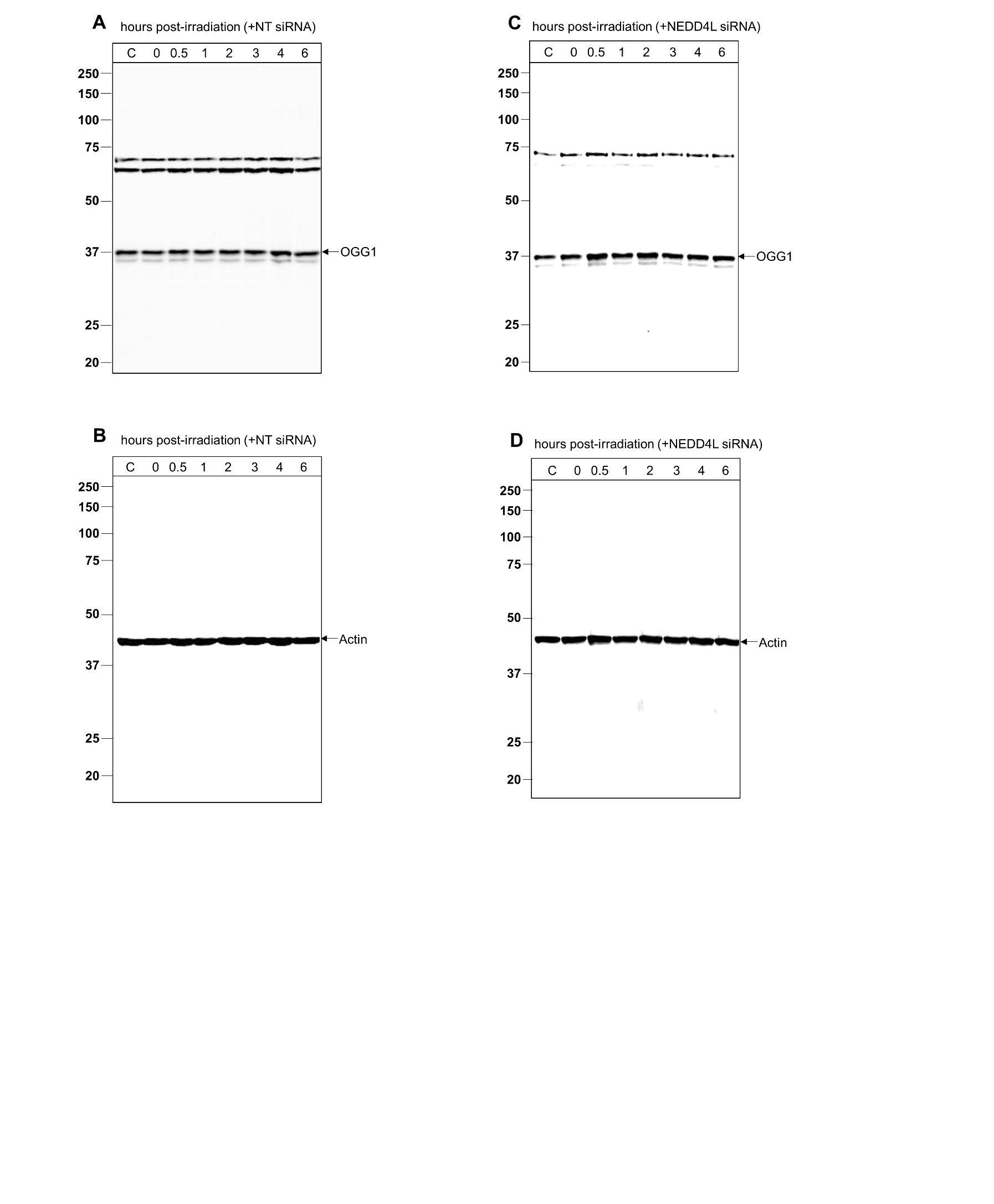
**

**Supplementary Figure 7.** NEDD4L controls OGG1 protein levels in response to DNA damage. (**A**-**D**) U2OS cells treated with (**A-B**) non-targeting (NT) control siRNA or (**C-D**) NEDD4L siRNA, and then were either unirradiated (C) or treated with x-ray irradiation (10 Gy) and harvested at the indicated time points post-treatment. Whole cell extracts were prepared and analyzed by SDS-PAGE and immunoblotting using (**A** and **C**) OGG1 antibodies, followed by (**B** and **D**) actin antibodies. Full length blots are shown.


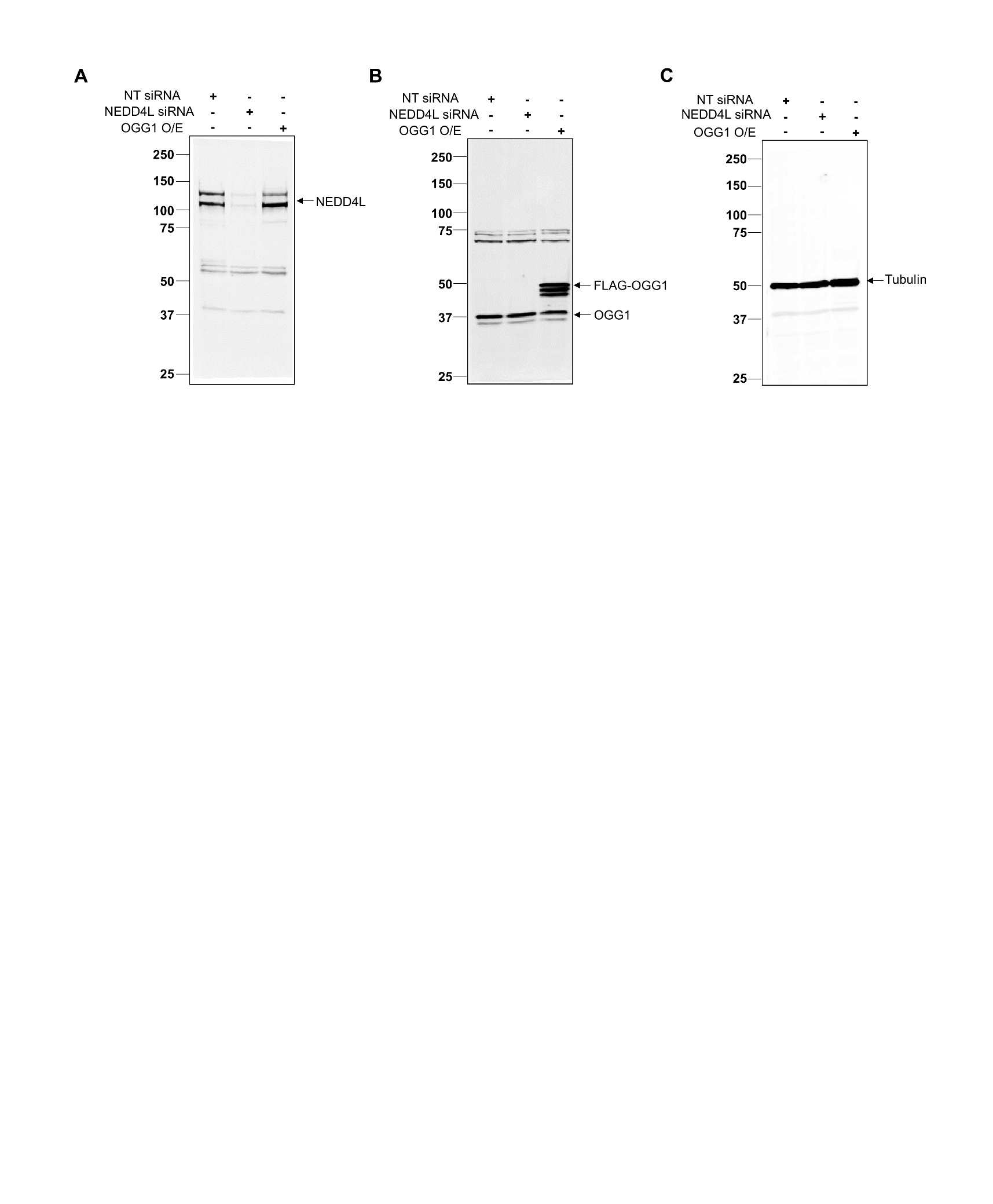


**Supplementary Figure 8.** Analysis of NEDD4L knockdown and OGG1 overexpression. U2OS cells were treated with NT control siRNA, NEDD4L siRNA, or following Flag-tagged OGG1 overexpression. WCE were prepared and analyzed by SDS-PAGE and immunoblotting using either (**A**) NEDD4L, (**B**) OGG1 or (**C**) tubulin antibodies. Full length blots are shown.
